# Efficient sequencing data compression and FPGA acceleration based on a two-step framework

**DOI:** 10.3389/fgene.2023.1260531

**Published:** 2023-09-21

**Authors:** Shifu Chen, Yaru Chen, Zhouyang Wang, Wenjian Qin, Jing Zhang, Heera Nand, Jishuai Zhang, Jun Li, Xiaoni Zhang, Xiaoming Liang, Mingyan Xu

**Affiliations:** ^1^ HaploX Biotechnology, Shenzhen, Guangdong, China; ^2^ Shenzhen Institutes of Advanced Technology, Chinese Academy of Sciences, Shenzhen, Guangdong, China; ^3^ Xilinx Inc., San Jose, CA, United States

**Keywords:** compression, fastq, sequencing, repaq, LZMA, FPGA acceleration

## Abstract

With the increasing throughput of modern sequencing instruments, the cost of storing and transmitting sequencing data has also increased dramatically. Although many tools have been developed to compress sequencing data, there is still a need to develop a compressor with a higher compression ratio. We present a two-step framework for compressing sequencing data in this paper. The first step is to repack original data into a binary stream, while the second step is to compress the stream with a LZMA encoder. We develop a new strategy to encode the original file into a LZMA highly compressed stream. In addition an FPGA-accelerated of LZMA was implemented to speedup the second step. As a demonstration, we present repaq as a lossless non-reference compressor of FASTQ format files. We introduced a multifile redundancy elimination method, which is very useful for compressing paired-end sequencing data. According to our test results, the compression ratio of repaq is much higher than other FASTQ compressors. For some deep sequencing data, the compression ratio of repaq can be higher than 25, almost four times of Gzip. The framework presented in this paper can also be applied to develop new tools for compressing other sequencing data. The open-source code of repaq is available at: https://github.com/OpenGene/repaq.

## Introduction

In recent years, with the increasing throughput of next-generation sequencers and the decreasing cost of per base sequencing, the amount of sequencing data has shown explosive growth. For example, the Illumina NovaSeq 6000 can generate more than 6T bases per run within 40 h, which means about 15T bytes of raw text files. Unlike the rapid decline in sequencing costs, the cost of data storage has fallen very slowly. Therefore, this large amount of sequencing data will bring high storage costs. Although the analysis result files (i.e., VCF files) can be much smaller than the raw data, the original raw data still have to be stored, in case the data needs to be reanalyzed. Especially for sequencing data generated in clinical medical applications (Liu et al., 2018; Priestley et al., 2019), storage of raw sequencing data is not only a consideration of data value, but also a legal requirement.

On the other hand, the increasing popularity of cloud computing makes the need for data transmission to the cloud particularly urgent. Next-generation sequencing data, due to its large size, was considered not suitable for being analyzed on the cloud. In recent years, the increase of public network bandwidth and the application of private networks have made it possible to transfer sequencing data to the cloud. However, the cost of network bandwidth to transfer sequencing data is still huge, and the data transfer time also needs to be greatly reduced.

Based on the above two reasons, it is imperative to efficiently compress the sequencing data. For the same reasons, many researchers have already studied the problem of sequencing data compression, and have developed a lot of algorithms and tools. These algorithms can be divided into lossy or lossless. Lossless algorithms are preferred due to sequencing has now widely applied in medical applications, which require data to be stored as losslessly as possible. For example, Sebastian et al. presented a specialized compression algorithm GSQZ for FASTQ data, and implemented DSRC based on this algorithm (Deorowicz and Grabowski, 2011). This tool was updated to DSRC2 to provide higher compression speed and better programming interfaces (Roguski and Deorowicz, 2014). FQC was presented as a novel approach for not only efficient compression, but also archival and dissemination of FASTQ datasets (Dutta et al., 2015). Marius et al. presented LFQC as a lossless FASTQ compressor (Nicolae et al., 2015), and demonstrated that it achieved better compression ratio on LS454 and SOLiD datasets. Sultan et al. presented LFastqC which has a better compression and decompression speed than LFQC (Al Yami and Huang, 2019). But on the most important Illumina (SOLEXA) dataset, both LFQC and LfastqC results were not optimal. Mark Howison developed a tool called SeqDB (Howison, 2013), which combined the existing multithreaded Blosc compressor with a new data-parallel byte-packing scheme.

For the compression of FASTQ files, the key components to compress are sequence and quality. Because individual genomes differ little within the same species, many researchers have proposed reference-based compression algorithms, in which the sequence strings in FASTQ files are aligned to reference genome and the alignment results are stored instead of the original sequences. For instance, Markus et al. presented a tool for efficient storage of sequencing data using reference-based compression (Hsi-Yang et al., 2011). This tool only works for resequencing data that targets well-studied genomes (i.e., *Homo Sapiens*). Since the sequence alignment is usually time-consuming, reference-based compression algorithms are usually slower than non-reference-based algorithms. Yongpeng et al. developed FQZip as a lossless reference-based FASTQ com-pressor (Zhang et al., 2015a), and showed that the speed can be improved by introducing a light-weight mapping algorithm (Zhang et al., 2015b). Most reference-based algorithms require aligning the sequences to reference genome completely. Different from this, an algorithm that applied cascading bloom filters was introduced to circumvent the need for complete alignment (Rozov et al., 2014). This algorithm could be much faster than other reference-based algorithms. However, the alignment was not concise and the compression ratio of this tool was a bit lower, especially for low coverage data (i.e., depth <50x).

Compression of quality values is also a focus of researchers. Raymond et al. compared different compression policies for quality scores, and found both lossy and lossless transformations were useful (Wan et al., 2012).

Most algorithms proposed to compress FASTQ quality scores were lossy compression methods (Cánovas et al., 2014; Malysa et al., 2015; Fu and Dong, 2017; Suaste and Houghten, 2021). The effect of lossy compression of quality scores on variant calling was also studied (Ochoa et al., 2016), and showed that the lossy compression can maintain variant calling performance comparable to that with the original data. Another study even reported a positive side effect that the lossy compression could unexpectedly improve genotyping accuracy (Greenfield et al., 2016). Since quality scores are considered as relatively less important, modern sequencers tend to apply simpler quality scoring schemes. For example, the data generated by Illumina NovaSeq have only 6 different quality bins, and most bases share the same quality score. In this case, lossless compression can almost achieve comparable compression ratio as lossy compression.

Besides compression of FASTQ files, some tools can also handle compression of alignment files in SAM/BAM format (Li et al., 2009). For example, samcomp was introduced as a SAM format compressor, whose performance was better than most competitors (Bonfield and Mahoney, 2013). Some new formats to store SAM format data were also proposed, such like CSAM (Canovas et al., 2016). The compression of aligned data is similar as reference-based of FASTQ data.

Data compression algorithms need to find a balance among compression rate, compression time, and memory consumption. Since modern computer systems usually have enough memory, the major problem is to obtain a high compression ratio within an acceptable time. Although dozens of tools have been developed for compressing sequencing data, there is still a strong need to develop a tool with a higher compression ratio. After deep investigation, we found that there is still potential that has not been tapped by previous studies. For instance, most of data are generated by paired-end (PE) sequencing technology (Campbell et al., 2008). In this case, the read pairs usually have overlapped region that are sequenced and stored twice (Chen et al., 2017), and this redundant information can be eliminated to improve compression ratio. The scheme of quality scoring has also changed a lot that only a few bins are used for modern sequencers like Illumina NovaSeq. New algorithms can be developed to efficiently compress such quality scores with a fast transformation. Existing tools also rarely utilized Lempel–Ziv–Markov chain algorithm (LZMA) for secondary compression since LZMA compression is usually slow. However, we found that if the original data could be repacked to a much small binary file, the secondary LZMA compression could be completed within an acceptable time.

In this paper, we present a two-step framework for compressing sequencing data. In the first step, a carefully designed algorithm can repack the original text files into a binary stream quickly, which is usually much smaller than a Gzip stream. Importantly, this stream can be further compressed by LZMA, while Gzip streams can hardly be recompressed by LZMA. The second step is to encode the repacked binary stream using a multi-threaded LZMA encoder. For demonstration, we introduce repaq, as a lossless non-reference FASTQ compressor based on such design. Different from all previously introduced tools, repaq applies read assembly to eliminate the redundancy of each read pair for paired-end sequencing data. We compared the performance of repaq and major other FASTQ compressors, and the result showed repaq offered a much higher compression ratio, while its speed remained comparable to Gzip and Bzip2.

LZMA compression is usually slow because the throughput in single-threaded software-based (CPU) implementation is limited to ∼2 MB/s. To achieve fast compression, we also implemented LZMA on Field Programmable Gate Arrays (FPGA). Although some previous studies already reported FPGA-based LZMA acceleration (Li et al., 2014; Bing et al., 2015; Zhao and Li, 2017), our FPGA implementation utilizes the features of new FPGA hardware and has advantages in speed and compression ratio. We used Xilinx SDx (C/C++/HLS) to implement high speed LZMA, and we achieved ∼105 MB/s throughput for compressing original FASTQ data on a Xilinx U200 FPGA device.

## Materials and methods

### Two-step framework for sequencing data compression

**FIGURE 1:**
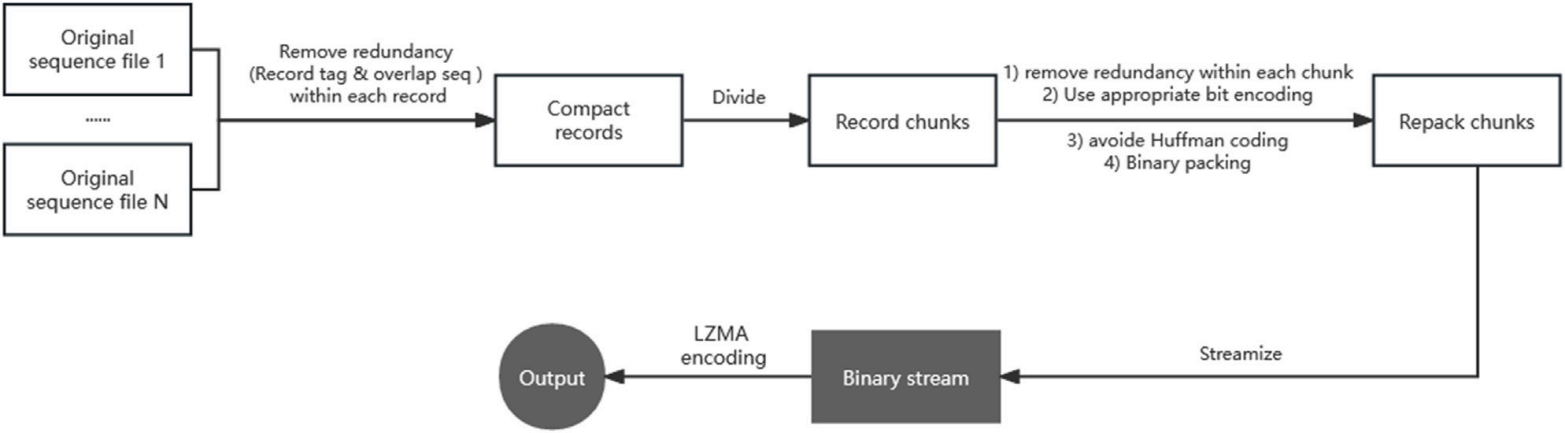
The two-step compression framework for sequencing data. Step 1/2 operations are marked with a white/gray background respectively. Removing redundancy is implemented in two major operations. An operation consists of removing redundancy (i.e., the same record tag and overlap sequence) between multiple files of one record. The other operation consists of removing redundancy (i.e., sequencer chip ID) between multiple records of one chunk. Using appropriate bit encoding greatly reduce the amount of data, Avoiding any Huffman coding is the most critical strategy in the bit encoding.

As the design of this framework can be applied to data compression in other formats, we will first introduce the framework briefly, and then present the algorithm and implementation details of repaq. The two steps of this compression framework are repacking and LZMA compression. Figure 1 gives a brief diagram of this two-step framework.

As shown in Figure 1, the first operation is to remove redundancy within each record. Sequencing raw data generally has multiple files, and these files are arranged by records. For example, paired-end next-generation sequencing FASTQ data can have two files (R1 and R2), and a record (read pair) consists of read1 and read2 from R1 and R2 respectively. Read1 and read2 of the same record share the same tag information (i.e., the sequencer chip ID, coordinates, sample barcodes), but are stored twice. Also if the insert size is shorter than the pair-end sequencing length, then there are sequences being recorded repeatedly in multiple files. To improve the compression ratio, our suggestion is to compress the data by record, rather than compressing each file independently.

The second operation is to divide data to chunks, which means sequentially splitting the file by records. Repaq has a parameter k to set chunk size. The default k is 1000 which means 1000 kilo bases. According to the characteristics of the data, a chunk can have hundreds to millions of records. Comparing to the whole data, a small chunk usually has a higher degree of similarity, which means that a greater percentage of common information can be extracted. The other purpose of this operation is to streamize the repacked output. When a chunk is repacked, the output data can be immediately passed to the LZMA compressor.

The key operation of the entire framework is to remove redundancy between multiple records of one chunk. For sequencing data, some information is duplicated (i.e., the sequencer chip ID, sample barcodes), and can be extracted and stored just once at the head of chunk. Some information is sequentially increasing or decreasing (i.e., the coordinates in the Meta information). It is appropriate to encode them with a baseline and a list of progressive difference. Most importantly, the alphabet of sequence information usually has only a few letters. Using appropriate bit encoding to convert sequence text into binary information can greatly reduce the amount of data. The most critical strat-egy is avoiding any Huffman coding (Knuth, 1985) in the bit encoding. LZMA algorithm uses a dictionary compression scheme, which searches for duplicated string byte by byte. Since the Huffman coding can result in character being represented by crossbyte bits, it will break the LZMA’s dictionary search algorithm and consequently generate an output that can hardly be further compressed by LZMA. For example, a Gzip-compressed file is almost not compressible by LZMA.

The last operation of this framework is to compress the binary stream using LZMA algorithm. Although there are some storage formats (i.e., CRAM) that use LZMA as their built-in compressor, LZMA is not widely adopted for compressing sequencing or genomic data due to LZMA being too slow to compress such large data. To make the LZMA compression acceptable in time consumption, the key is to make the output of the first step as small as possible. With the expense of compression ratio, multi-threaded parallelization can make LZMA run faster. Since sequencing files are usually very big, increasing the dictionary size of LZMA can usually improve the compression ratio greatly.

### Repaq: a lossless FASTQ compressor with ultra-high compression ratio

Based on above two-step compression framework, repaq is implemented as a non-reference-based lossless FASTQ compressor. This tool accepts single-end or paired-end next-generation sequencing data as input, and outputs repacked FASTQ data (with.rfq as extension) or LZMA-compressed repacked FASTQ data (with.rfq.xz as extension).

**FIGURE 2:**
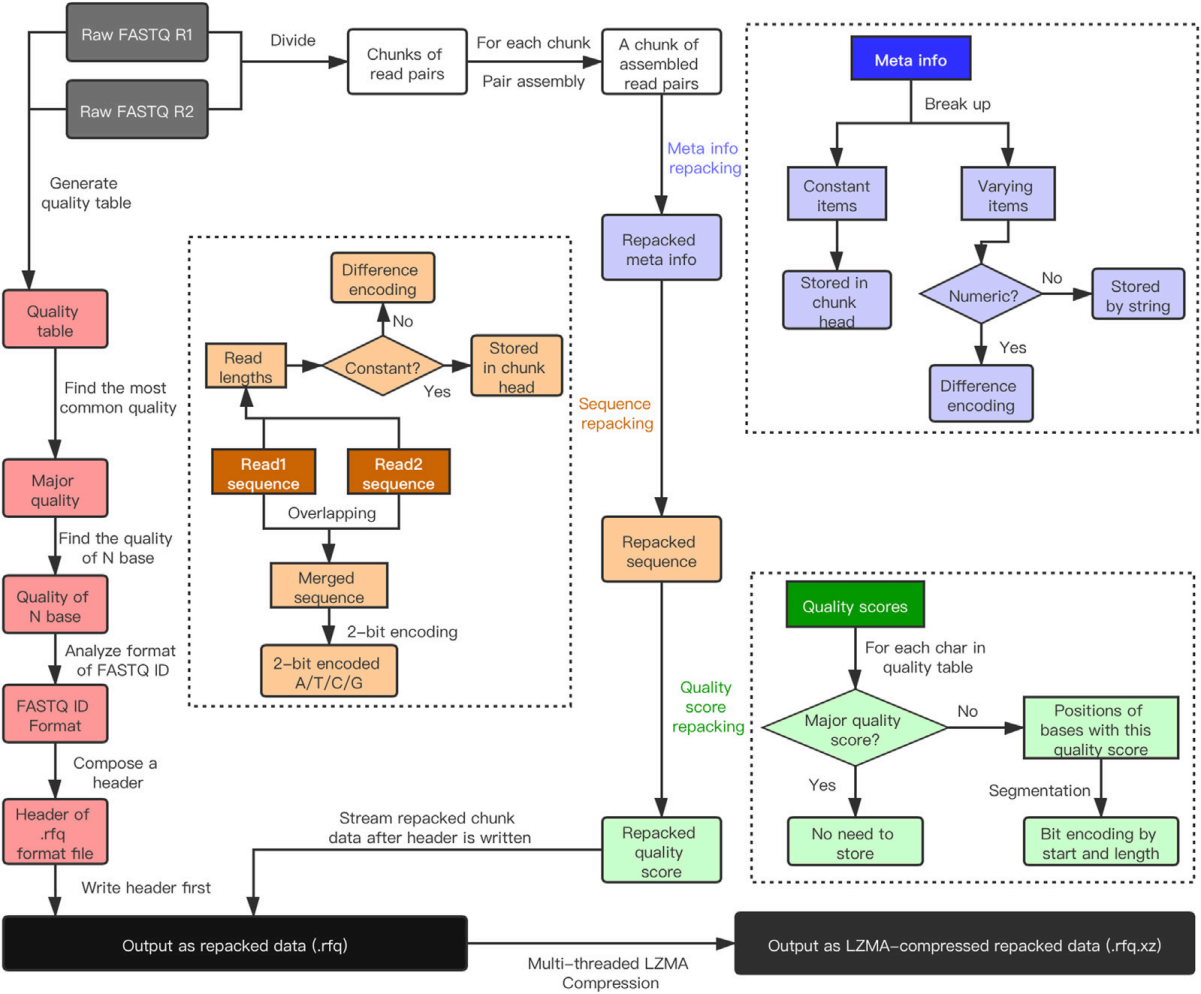
The repaq workflow. The input files are a pair of PE FASTQ, while the output can be repacked data (.rfq) or LZMA-compressed repacked data (.rfq.xz). The workflow can be briefly divided into five parts: header generation (red), Meta information repacking (blue), sequence repacking (brown), quality score repacking (green) and LZMA compression.

As shown in Figure 2, the input FASTQ files are first divided into read pair chunks, and then each chunk will be repacked by repacking Meta info, sequence and quality scores separately. Before the first chunk is encoded and written, the input files are partially read and analyzed to generate a header. Besides the paired-end FASTQ, repaq also accepts single-end FASTQ as input. However, if the data was generated by paired-end sequencing technology, it is recommended to compress them together to obtain higher compression ratio.

### Header generation

Repaq traverses the first chunk to generate the header. In this operation, the quality table is gathered. The most common quality score in the quality table is marked as major quality score. Typically all the N bases share the same quality score. In this case, this quality score is marked as N-score. Once an N-score is recorded, there will be no need to store N bases, since they can be recovered by the positions of N-scores. The identifiers of this FASTQ are also parsed to get the sequencing file format, which will be stored in the header as flags. The algorithm version is also stored in the header for software backward compatibility.

### Meta info repacking

Meta information is stored in the FASTQ identifiers. After traversing all identifiers of one chunk, repaq extracts the constant items and separates the varying items. The constant items are stored in the chunk head, whereas the varying items are encoded in other ways. If a varying item is numeric (i.e., the coordinates of lane, X, Y), it will be encoded by progressive difference. Otherwise it will be stored directly.

### Sequence repacking

The sequences of a chunk are concatenated, and then encoded by 2-bit for A/T/C/G bases. As mentioned above, if N-score is found and is consistent in this chunk, the N bases will be not recorded. Otherwise, the positions of N bases will be stored for this chunk. If the input data are paired-end, the read1 sequence and read2 sequence will be assembled by detecting their overlapping region. The merged sequence will be stored instead of storing them separately to reduce redundancy. Since all sequences are concatenated together, the read lengths are also need to be stored. If the sequence lengths are not constant, each length will be encoded by one or several bytes, depends on the bytes needed to represent the maximum length.

### Quality score repacking

Repaq implements two different quality score repacking strategies. The first strategy, which is also illustrated in Figure 2, is called column-based encoding. In this strategy, positions for each quality score are segmented to many segments of consecutive positions. The progressive offset of the *k*th segment OFFSETk, which means POSk—POSk-1, as well as the segment length LENk, are stored. The combo < OFFSETk, LENk > is stored in one to 4 bytes adaptively, depends on how many bytes are needed to represent it. The second strategy is called run-length coding. In this strategy, all quality scores are stored interleaved. And the combo < SCOREk, LENk >, which is encoded in 1 byte, is used to store each segment. Depending on how often each quality value appears, the bits used to represent a score in the quality table is different. The more common quality scores, the fewer bits are used, so that the remained bits can be used to represent longer LENk. Repaq chooses the first strategy if the number of quality bins is less than 64, which is mostly true for modern sequencing data.

### LZMA compression

The repacked data is already compressed, and can be output directly as an.rfq file. The repacking process is ultra-fast and memory-efficient, while offering much higher compression ratio than Gzip and Bzip2. However, this repacked data can be further compressed by LZMA, and output as an.rfq.xz file. This is achieved by calling a multi-threaded LZMA compressor. In most cases, the LZMA-compressed repacked data is as small as 40% of the repacked data.

### FPGA implementation of LZMA

**FIGURE 3:**
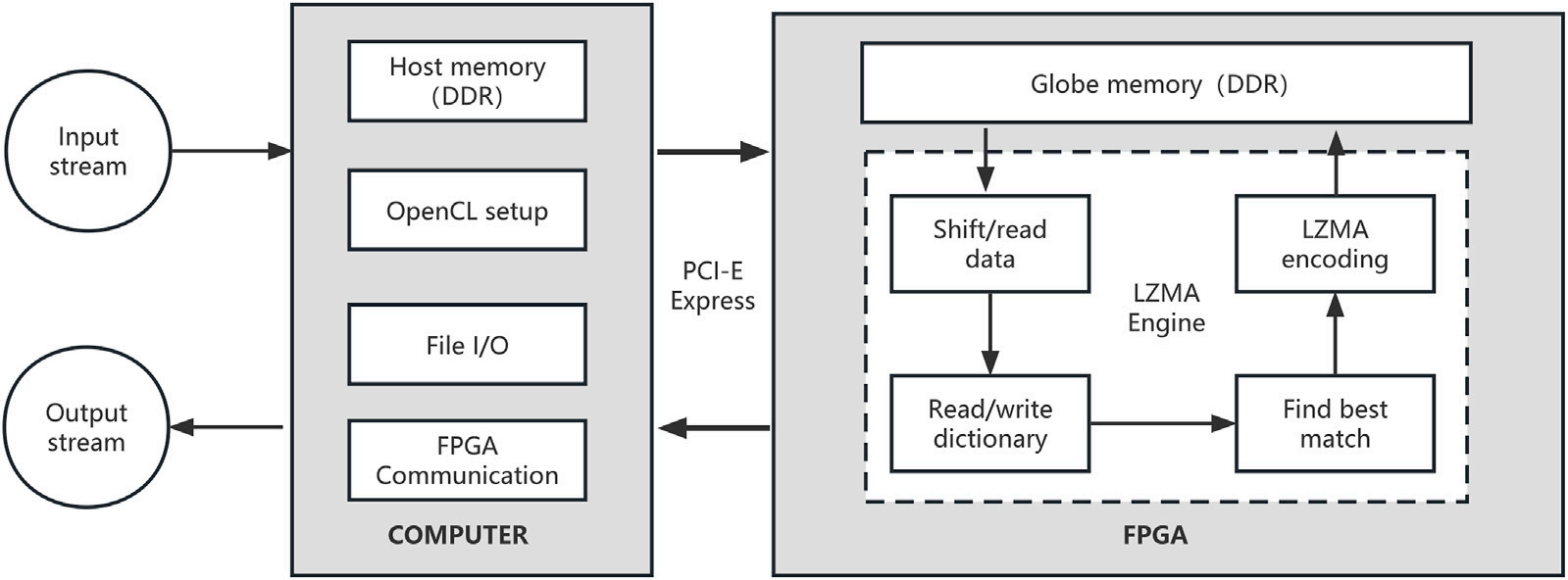
The workflow of FPGA-based LZMA. The most computationally intensive part is executed on the FPGA device. A LZMA engine is executing on the FPGA device, which is responsible for data accessing, dictionary searching and LZMA encoding.

LZMA application is derived from a byte-oriented compression scheme Limpel-ziv (LZ). We use FPGA to accelerate LZMA compression to further improve the compression speed of repaq. The FPGA-based LZMA has computer part and FPGA part. The computer coordinates the FPGA device setup and communication, while main LZMA compression algorithm is executed on FPGA device. The workflow of FPGA-based LZMA is described in Figure 3.

In the global memory on FPGA device, several 4 GB dictionaries are used to store the hash-indexed historical data. String keys of fixed length k (k = 11 as default) will be searched in the dictionaries. The string will be extended until a mismatched byte is found. The matching result will be represented as 64-bit data, which will be encoded to a LZMA stream. In our implementation, we used four dictionaries, and the max length of a match was limited to 256.

## Results

**FIGURE 4:**
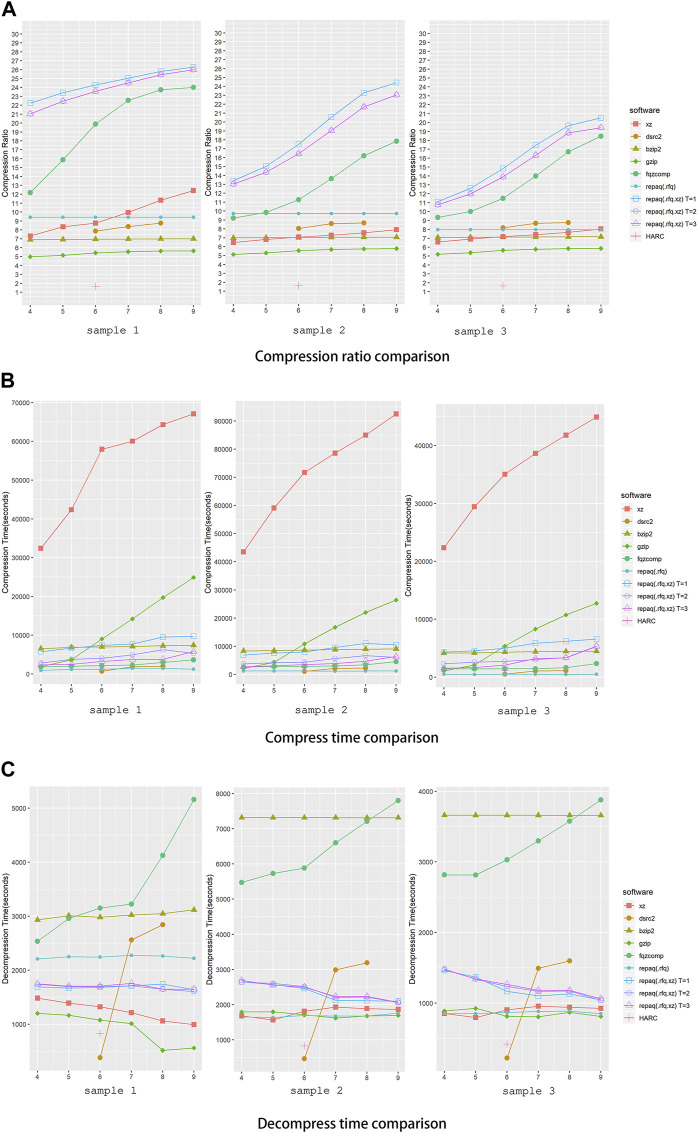
Performance comparison of repaq and other tools. **(A)** Shows the comparison ratio of comparison, **(B)** shows the compress time of comparison, while **(C)** shows the comparison of time to decompress. Three samples were tested in this experiment. *X*-axis indicates compression level setting, while *Y*-axis indicates compression ratio. The DSRC2 has only three compression levels (0,1,2), so it is aligned to the middle compression level (6,7,8) in the figure. Sample 1 is a paired-end deep target cap-turing data (39 GB + 39 GB), while sample 2 is a paired-end whole exome sequencing data (46 GB + 46 GB) and sample 3 is a single-end whole exome sequencing data (46 GB). For repaq, both non-LZMA mode (.rfq) and LZMA mode (.rfq.xz) mode were tested. For LZMA mode repaq, different threading setting were applied (T = 1, 2, 3).

To evaluate the performance of repaq, we conducted an experiment to compare repaq against other general or FASTQ specific compression tools. Gzip, Bzip2 and XZ were selected as general compression tools to compare since they are most used. Gzip is based on the DEFLATE algorithm, which is a combination of LZ77 and Huffman coding (Knuth, 1985), whereas Bzip2 is based on Burrows-Wheeler algorithm (Manzini, 2001) and XZ is based on LZMA. Fqzcomp (Bonfield and Mahoney, 2013), HARC (Chandak et al., 2018) and DSRC2 (Roguski and Deorowicz, 2014) were selected as FASTQ specific compressors to compare since they are widely discussed and cited, and are known as FASTQ compressors with good performance. Experiments were conducted on a system with Intel Xeon E5-2699 V4 CPU (55M Cache, 2.20 GHz) and 512G RAM. The compression ratio, compress time and decompress time were recorded, and the result is shown in Figure 4.

As shown in Figure 4A, LZMA mode repaq achieved much higher compression ratio than all other tools. The best compression ratio was achieved by repaq with single thread (T = 1), while repaq with two (T = 2) and three (T = 3) threads achieved almost same compression ratio. The tool achieved closest performance to repaq is fqzcomp, whose compression ratio is still about 20% lower than repaq. It should be noted that HARC only compresses the sequences, and does not compress the quality scores. This might be the major reason that HARC achieved worst overall compression ratio.

From Figures 4B, C, we can learn that LZMA mode repaq has comparable compress time and decompress time against other tools. Even with single thread applied, LZMA mode repaq is faster than Gzip when compression level is greater than 5. Comparing to LZMA mode repaq, fqzcomp takes less time to compress, but takes much more time to decompress. DSRC2 performs well in terms of compression time and decompression time, but its compression ratio compression ratio is several times lower. It is important to note that all decompression files have no information loss relevant to original file.

To evaluate the performance in Illumina short-read sequencing data and long-read sequencing data produced on PacBio SMRT and Oxford Nanopore platforms, we conducted another experiment to compare repaq against SPRING, which supports lossless compression and long read compression (Chandak et al., 2019). First, we used a range of different types of datasets, including well-known benchmark dataset SRR12300962, SRR12048570 and paired-end whole exome sequencing data (100G+100G). We then separately compressed these datasets using repaq and SPRING in single thread. Table 1 presents statistical information on the compression ratio at the highest compression level of 9. Both SPRING and repaq can achieve similar compression ratio. However, in terms of time consumption, SPRING took approximately 2.37 times longer than repaq in handling Illumina data. Repaq did not have an advantage in terms of compression time when dealing with long-read sequencing data, but the compression rate is better or extremely close to SPRING.

**TABLE 1:** Compression ratio for different platform datasets.

Sample	Platform	File size (Gb)	SPRING	Repaq
Sample4	Illumina	100 + 100	21.28	21.43
SRR12048570	PacBio SMRT	155	2.67	2.83
SRR12300962_1	Oxford Nanopore	56	2.24	2.22

It is worth mentioning that the non-LZMA repaq, which is ultra-fast due to no LZMA encoding applied, achieved higher compression ratio than Gzip and Bzip2. And for paired-end mode, it also outperformed DSRC2. Therefore, for the applications that compress and decompress time are critical, non-LZMA repaq can be an excellent alter-native to Gzip, Bzip2 and DSRC2, etc.

It is worth noting that Gzip/LZ77 compression is much ineffective for the stream of packaged record chunks, while LZMA can effectively further compress it. This reveals the potent combination of byte repacking and LZMA compression, offering an effective strategy to tackle compression issues for various data types. Our analysis underscores the importance of byte alignment, which requires each element to be repacked to either a section of a single byte or multiple complete bytes, for optimal LZMA compression. Within the process, quality score repacking and sequence repacking play a significant role in enhancing the compression rate, whereas LZMA compression chiefly affects time consumption.

### Performance of FPGA-based repaq

**FIGURE 5:**
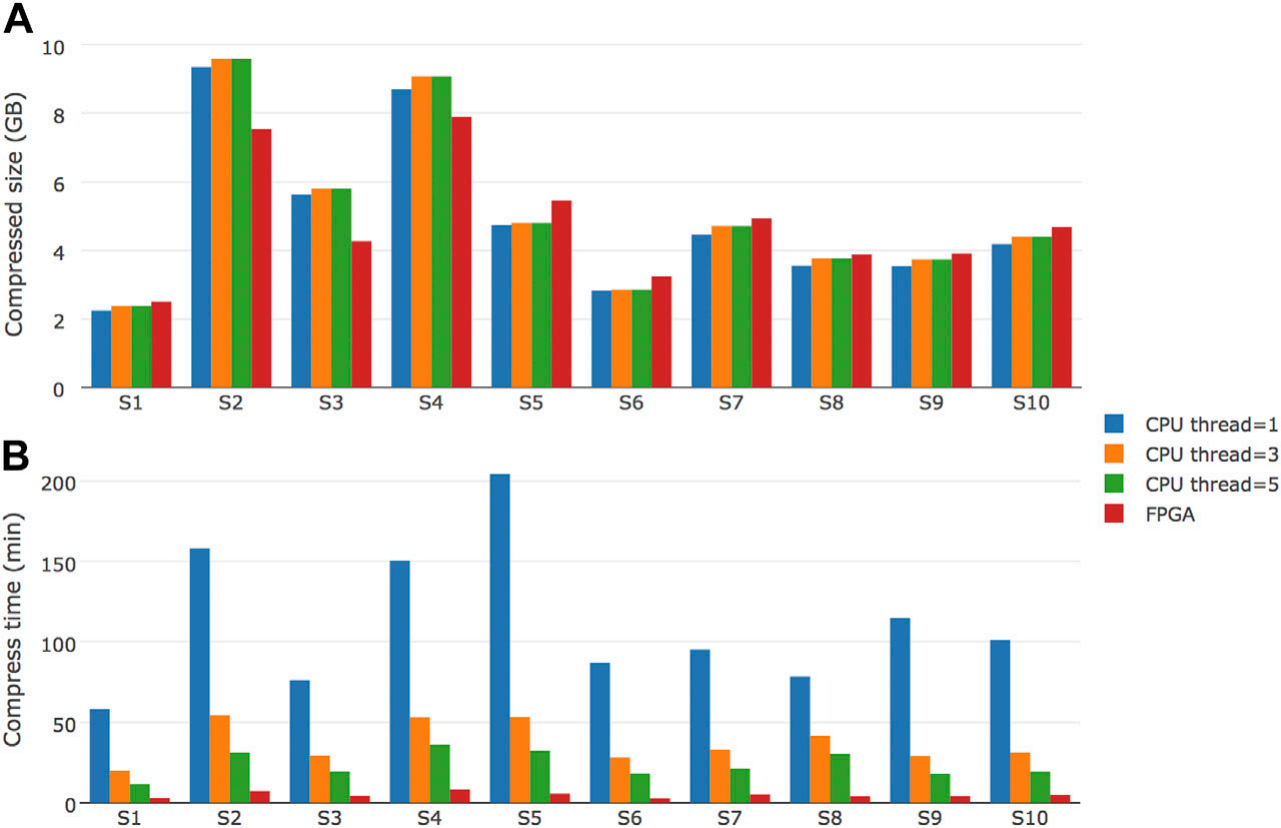
Performance comparison of FPGA-based repaq and CPU-based repaq. Ten samples were tested, with compressed file size and time to compress recorded **(A)** Shows the comparison of compressed file size in Gigabytes, while **(B)** shows the comparison of time to compress in minutes.

To evaluate the performance of FPGA-based repaq implementation, we tested a total of 10 files on a system with Intel Xeon E5-2699 V4 CPU (55M Cache, 2.20 GHz) and a Xilinx U200 FPGA device. The result is shown in Figure 5.

From Figure 5A, we can learn that FPGA-based repaq generated slightly bigger compressed files for 7 of 10 samples, but generaetd much smaller compressed files for the other 3 samples (S2, S3 and S4). From Figure 5B, we can find the FPGA-based repaq is much faster than CPU-based repaq. Even with 5 threads, the CPU-based repaq takes much more time than FPGA-based repaq. Specifically, the FPGA-based repaq achieved about 105 MB/s compression throughput for original FASTQ data. This makes realtime compression of FASTQ data possible, which can be much useful for building modern FASTQ storage engines.

## Conclusion and future work

In this paper, we introduced a two-step framework for compressing sequencing data, and demonstrated a novel FASTQ compressor repaq. The performance evaluation result showed that repaq has a compression rate far superior to other tools, while it takes comparable time to compress and decompress. We also introduced the FPGA-based acceleration of repaq, and demonstrated that the FPGA-based version is much faster than CPU-based, while the compression ratio remains comparable with CPU-based version. It is worth mentioning tha, our FPGA-based acceleration algorithm can be used for accelerating any general LZMA-based compression algorithm.

Repaq is an open-sourced and industry-oriented FASTQ compression tool. It can be deployed on a local cluster or in the cloud. Repaq has already adopted by some institutions that produce and process large amount of FASTQ data. In the author’s institution, repaq compresses tens of terabytes of data every day. Repaq demonstrates clear advantages in both compression ratio and compression time when handling short-read data with high sequencing depth. However, its performance may slightly diminish when dealing with long-read or low-depth sequencing data.

Although repaq has evolved to be a FASTQ compressor with industry strength, we still think that there is still a lot of work to be done in the future. Our primary plan is to reduce the compression time in long-read sequencing data. We also plan to implement a compressor for long-read sequencing data, such like data generated by PacBio Sequel and Oxford Nanopore platforms. For instance, the data generated by PacBio sequencers are usually stored in HDF5 format (Mason et al., 2010; Folk et al., 2011), which is usually highly redundant. Although there exist some tools can compress general HDF5 format files or convert them to BAM format files (Yu et al., 2017), it is preferred to develop a specific compression tool for compressing HDF5-formated long read sequencing data. In addition, currently the LZMA mode repaq is not memory efficient since the backend LZMA compressor consumes too much memory. Reducing memory consumption will be one of the future optimization directions of the algorithm.

In summary, as the sequencing data becomes larger and larger, efficient data compression will become increasingly important. The repaq tool can greatly compress FASTQ files, and has great value in applications such as archiving data or transferring data to cloud. The FPGA-based LZMA acceleration can used to accelerate repaq and other LZMA-based compression algorithms. Furthermore, the two-step compression framework proposed in this paper can help developing new sequencing data compression algorithms.

## Data Availability

The data analyzed in this study is subject to the following licenses/restrictions: Due to local restrictions on the sharing of biological data, the data generated/analyzed from this study is available via corresponding authors upon request. Requests to access these datasets should be directed to chen@haplox.com.
